# A Rare Case of Soft-Tissue Infection Caused by *Raoultella planticola*

**DOI:** 10.1155/2010/134086

**Published:** 2010-08-01

**Authors:** Karina O' Connell, Jack Kelly, Una NiRiain

**Affiliations:** ^1^Department of Medical Microbiology, University College Hospital, Galway, Ireland; ^2^Department of Plastic Surgery, University College Hospital, Galway, Ireland

## Abstract

*Raoultella* species are Gram-negative, non-motile bacilli primarily considered to be environmental bacteria. *Raoultella planticola* is a rare cause of human infections. We report a case of serious soft-tissue infection in a young male tiler who presented with cellulitis of his left thumb. He had sustained a crush injury to his left thumb 10 days earlier in a soiled environment. He noted a minor break in the skin and he washed the wound out with running water. One week later, he experienced pain, erythema, and swelling of his thumb and attended his general practitioner who prescribed oral flucloxacillin and penicillin V. Despite this treatment, he noticed progressive erythema and swelling of his thumb requiring hospital admission 3 days later. He underwent washout and debridement of his thumb. Tissue obtained intraoperatively cultured *Raoultella planticola*. He was treated with broad-spectrum antibiotics including ciprofloxacin and made a full and rapid recovery.

## 1. Introduction

*Raoultella* species are Gram-negative, non-motile bacilli primarily considered to be environmental bacteria. Although *Raoultella planticola* has only been identified on four occasions as the cause of human infections, there are many reports of its presence in clinical specimens. We report a case of serious soft-tissue infection caused by *R. planticola* in a young man who made a full and rapid recovery. To our knowledge, this is the first reported case of soft-tissue infection caused by this organism. 

## 2. Case Report

On the 2nd of March, 2010, a 30-year-old male was admitted to a tertiary referral hospital in the west of Ireland with a cellulitis of his left thumb and ascending lymphangitis. He had no previous medical history and was not on any regular medications. He worked as a tiler and had sustained a crush injury to his left thumb from a hammer ten days earlier. He described the floor on which he was working as being in poor condition and very soiled. He reported a minor break in the skin and washed his thumb with running water only. Three days prior to admission, he had developed painful swelling and erythema of his left thumb. He attended his general practitioner who prescribed oral flucloxacillin and penicillin V. His symptoms got progressively worse and on arrival to the Emergency Department he was systemically unwell with a temperature of 38.0°C, malaise, and chills. His blood pressure, respiratory rate, and heart rate were all within normal parameters.

On examination, the patient had a tense swelling of the left thumb with cellulitis extending to his left hand and forearm with associated ascending lymphangitis to the left axilla. He was commenced on high-dose intravenous benzyl penicillin 2.4 g QDS, flucloxacillin 2 g QDS, and clindamycin 600 mg QDS to cover for a clinically suggestive Group A streptococcal infection with additional staphylococcal cover. He was taken to theatre on the day of admission where he underwent washout and debridement of his left thumb. A tissue specimen and two intraoperative swabs were sent to the Microbiology Department for Gram staining and culture. 

Gram-negative bacilli were seen on the Gram stain of the tissue specimen obtained in theatre, and a Gram-negative organism was subsequently cultured from this specimen as well as from the two swabs taken intraoperatively. Methicillin-sensitive *Staphylococcus aureus* was also cultured from the pus swabs, but not from the tissue specimen. Anaerobic cultures were negative. Ciprofloxacin was added to the antimicrobial regimen and the patient's cellulitis improved remarkably within a few days. The organism was identified as *Raoultella planticola* using the VITEK-2 biochemical identification system with a 93% probability. Antibiotic susceptibility testing was carried out and revealed resistance to amoxicillin but susceptibility to co-amoxiclav, ciprofloxacin, cephalosporins, and aminoglycosides. The patient received six days of intravenous antibiotics and was discharged home on oral antibiotics to complete a two-week course of antibiotics in total.

## 3. Discussion

This case describes a serious soft-tissue infection in a young male tiler ten days post crush injury with a hammer to his left thumb. The putative primary causative organism *R. planticola* has only very rarely been described in the medical literature as a cause of clinical infection in humans although it has been isolated several times from clinical specimens. Podschun et al. carried out a 1-year survey of newborns on a neonatal ward and noted that *K. planticola* accounted for 8.7% of the *Klebsiella *spp. recovered from oropharyngeal and rectal swabs [1]. 

The genus *Raoultella *was created in 2001 based on analysis of the sequences of the 16S rRNA and rpoB genes [2]. Until this time, the organism was identified as being a member of the *Klebsiella *genus, part of the family “Enterobacteriaceae”. It was originally described as *Klebsiella planticola* in 1981 and later in 1983 as *Klebsiella trevisanii *[3, 4]. In 1986, it was recommended that the name *K. planticola *be used for the species described by Bagley et al. and Ferragut et al. [3, 4]. *Raoultella planticola* was originally considered to be an environmental bacterium. The first case report of human infection was in a patient with septicaemia admitted to an intensive care unit in Lyon, France [5]. In 1986, Freney et al. identified two cases of septicaemia and 24 examples of patient colonisation by *Klebsiella trevisanii* in a study carried out over an 18-month period in Lyon [6]. The first case was a patient who experienced a bloodstream infection with *K. trevisanii* nine days post mitral valve replacement for infective endocarditis, and the second case was a patient with pneumonia and septicaemia ten days post coronary artery bypass graft. The sites of origin of the 24 strains of *Klebsiella trevisanii* that were not linked with infection were as follows: 14 from tracheal aspirate, 3 from urine, 2 from sputum, 2 from throat, 1 from cerebrospinal fluid, 1 from nose, and 1 from venous catheter. The authors concluded that the organism “appears to have little virulence for humans,” as both cases occurred in compromised hosts. The most recent case in the literature describes a 45-year-old man with pancreatitis and a retroperitoneal abscess [7]. *R. planticola* was cultured from the peritoneal fluid and from intraabdominal pyogenic specimens. The authors in this case felt that previous antimicrobial use may have been selected for the organism. 

In conclusion, the patient in this case experienced a serious soft-tissue infection with *R. planticola*, confirmed by culture of the organism from a tissue specimen. The organism was then identified using the VITEK-2 with a 93% probability. Evaluation of the VITEK-2 GN card for the identification of Gram-negative bacilli in 2005 revealed that the system correctly identified 97.4% of the 426 isolates tested [8]. To date, only four case reports of human infection with *R. planticola* have been described. To our knowledge, this is the first case report of a serious soft-tissue infection caused by *R. planticola.*
